# Unruptured Basilar Tip Aneurysm with Internal Septation: Coiling Implications?

**DOI:** 10.1155/2016/3697985

**Published:** 2016-11-06

**Authors:** Ayman Khalil, Hong Kuan Kok, Mark Schembri, Paul Brennan, Mohsen Javadpour, John Thornton, Alan O'Hare, Hamed Asadi

**Affiliations:** ^1^Department of Neurosurgery, Beaumont Hospital, Dublin 9, Ireland; ^2^Neurointerventional and Interventional Radiology Service, Department of Radiology, Beaumont Hospital, Dublin 9, Ireland

## Abstract

An internal septum within a basilar artery aneurysm is an infrequent anomaly and is very rarely reported in the literature. We report a 62-year-old lady that was incidentally diagnosed with basilar tip aneurysm. Further imaging with magnetic resonance imaging (MRI) revealed internal septation within this aneurysm which was later confirmed with digital subtraction angiography (DSA). She underwent coil embolisation, which involved technical manipulation of the microcatheter and the balloon to enable coiling of each separate aneurysm compartment. We present this case to illustrate the effect of this anatomical variation on the selection of endovascular treatment strategy.

## 1. Introduction

Lobulated basilar tip aneurysms are not uncommon; however, an internal septum is an infrequent anomaly and is very rarely reported in the literature and can sometimes be associated with basilar artery fenestration [1, 2].

In this case report, we describe an incidentally detected large basilar tip aneurysm with an internal septum, treated successfully with coil embolisation.

## 2. Case Report

**Figure 1:**
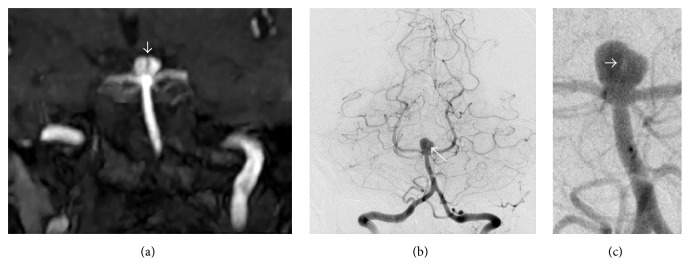
MRI demonstrating a saccular basilar tip aneurysm with a likely internal septum (a) which was confirmed on angiography (b) and its magnified view (c).

A 62-year-old lady presented with a 6-week history of intermittent dizziness and underwent magnetic resonance imaging (MRI) of the brain, revealing an 8 × 6 mm basilar tip aneurysm. On careful review of the MRI angiographic images, internal septation within this aneurysm was suspected (Figure 1(a)), arising from its dome extending into the neck. This finding was subsequently confirmed on digital subtraction angiogram (DSA) (Figures 1(b) and 1(c)).

Following multidisciplinary consensus and discussion with the patient, taking into account her age and aneurysm size, it was decided to treat this aneurysm by coil embolisation.

**Figure 2:**
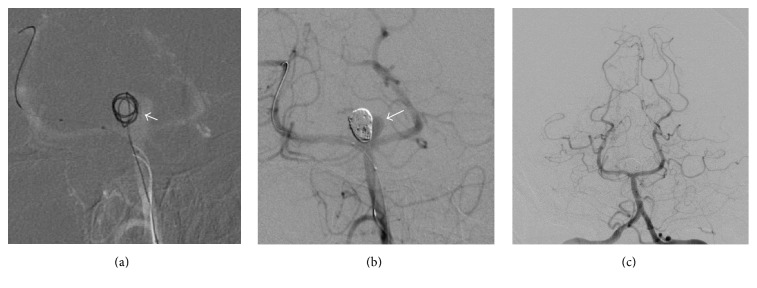
Microcatheter positioned within the right sided aneurysmal compartment, with the coil loops abutting the septum (a). Right sided chamber complete occlusion with patent left chamber (arrow) with coils constrained by the septum (b). Complete coil embolisation of both compartments (c).

Following right common femoral arterial access and through a 6-French left vertebral artery guide catheter, a 6 × 9 mm ECLIPSE™ balloon (BALT Extrusion, Montmorency, France) was initially placed across the aneurysm neck into the right P1 and an EXCELSIOR™-SL10 catheter (Stryker Neurovascular, Fremont, CA) was positioned into the right sided compartment of the aneurysm with the internal septum clearly visible, constraining the movements of the microcatheter tip (Figure 2(a)). Subsequently, multiple TARGET™ coils (Stryker Neurovascular) were used to pack the separate right sided chamber, subtly shifting the septum towards the left (Figure 2(b)). Subsequently, the balloon was repositioned into the contralateral left P1 and the microcatheter was also repositioned into the left sided compartment, successfully occluding it with multiple TARGET coils (Figure 2(c)).

There was immediate technical success with no peri- or postprocedural complication. A follow-up MR angiogram is planned in 6 months' time for assessment.

## 3. Discussion

At around the 5 to 9 mm fetal stage, the basilar artery is formed as a result of paired fetal longitudinal artery fusion [3], and partial failure of this process can result in basilar artery fenestration [4]. An explanation for the formation of compartmentalised basilar tip aneurysms is also hypothesised to be related to a similar phenomenon [1, 2]; however, to our knowledge, the exact incidence of this condition is unclear.

Bilobed basilar tip aneurysms are well described and frequently observed [5] on noninvasive as well as catheter angiographic images prior to intervention. Bilobed aneurysms can normally be treated as a single intrasaccular space as the lobulated indentation over the aneurysm dome does not usually extend into the aneurysm neck as a septum. Similarly, multiple adjacent aneurysms are usually also detected and characterised prior to intervention and are usually treated as separate aneurysms without any particular interference on each other's management.

Conversely, a septate aneurysm is difficult to recognise in advance, with potentially significant implications on the treatment strategy employed [6]. As an anatomic variant, it is likely to be inconsequential for surgical treatment by clipping [7]. However, it is important to recognise when an endovascular approach is being considered, particularly in the setting of acute rupture. One of the potential risks that could result from failure to recognise this variant is the overestimation of the aneurysm compartmental size and corresponding intrasaccular devices for occlusion.

Given the current evidence showing superiority of coil embolisation [8], awareness of this anatomical variation will influence the selection of specific endovascular devices such as bare or matrix coils and embolisation devices such as the WEB™ (Sequent Medical, Aliso Viejo, CA) or MEDINA™ (Medtronic, Dublin, Ireland) where deployment can be potentially complicated by the presence of the septum. In these situations, each compartment will likely require treatment as a separate aneurysm, with separate microcatheter cannulation, while being conscious of the possible detrimental effects of overpacking one compartment on the other. If such an approach is considered appropriate, implantation of two devices as described for WEB (Sequent Medical) in bilobed aneurysms can also be an option in this setting, either sequentially or concurrently [5].
